# Drug Nanorod-Mediated Intracellular Delivery of microRNA-101 for Self-sensitization via Autophagy Inhibition

**DOI:** 10.1007/s40820-019-0310-0

**Published:** 2019-09-25

**Authors:** Xiaofei Xin, Xiaoqing Du, Qingqing Xiao, Helena S. Azevedo, Wei He, Lifang Yin

**Affiliations:** 1 Department of Pharmaceutics, School of Pharmacy, China Pharmaceutical University, Nanjing, 210009 People’s Republic of China; 2 School of Engineering and Materials Science, Institute of Bioengineering, Queen Mary, University of London, London, E1 4NS UK

**Keywords:** Nanocrystals, MicroRNA delivery, Autophagy inhibition, Cytotoxicity, Combinatorial therapy

## Abstract

Autophagy is closely related to the drug resistance and metastasis in cancer therapy. Nanoparticle-mediated co-delivery of combinatorial therapy with small-molecular drugs and nucleic acids is promising to address drug resistance. Here, a drug-delivering-drug (DDD) platform consisting of anti-tumor-drug nanorods as a vehicle for cytosol delivery of nucleic acid (miR-101) with potent autophagic-inhibition activity is reported for combinatorial therapy. The developed 180-nm nanoplatform, with total drug loading of up to 66%, delivers miR-101 to cancer cells, with threefold increase in intracellular level compared to conventional gene carriers and inhibits the autophagy significantly, along with above twofold reduction in LC3II mRNA and approximately fivefold increase in p62 mRNA over the control demonstrated in the results *in vivo*. And in turn, the delivery of miR-101 potentiates the drug’s ability to kill cancer cells, with a threefold increase in apoptosis over that of chemotherapy alone. The anti-tumor study *in vivo* indicates the combined therapy that enables a reduction of 80% in tumor volume and > twofold increase in apoptosis than of the single-drug strategy. In summary, via the carrier-free strategy of DDD, this work provides a delivery platform that can be easily customized to overcome drug resistance and facilitates the delivery of combined therapy of small-molecular drugs and nucleic acids.

**Electronic supplementary material:**

The online version of this article (10.1007/s40820-019-0310-0) contains supplementary material, which is available to authorized users.

## Highlights

- Nanosized cytotoxic drug could combat against the autophagy induced by the drug.
- The drug nanorods enabled efficient intracellular delivery of the nucleic acid and the resultant autophagy inhibition in vitro and in vivo, which in turn sensitized the cancer cells to the anti-tumor nanorods.

**Electronic supplementary material:**

The online version of this article (10.1007/s40820-019-0310-0) contains supplementary material, which is available to authorized users.

## Graphical abstract

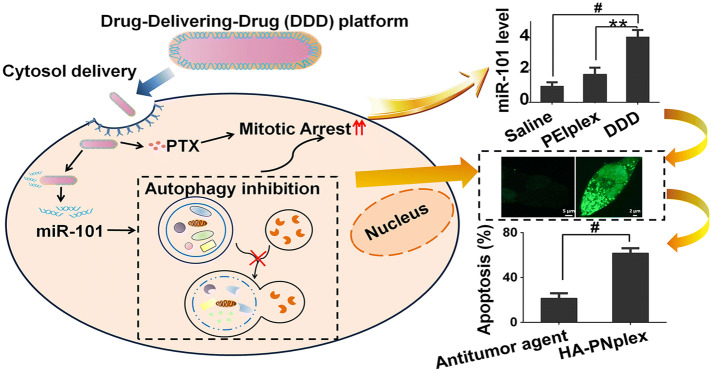

## Introduction

Despite the significant progress achieved in targeted therapies, the development of drug resistance has emerged as the next challenge for effective therapy in diverse diseases, such as tuberculosis, microbial infections and in particular cancer [1, 2]. For cancer chemotherapies, the major mechanisms of drug resistance include increased metabolic activation, elevated expression of the drug target, changed target or pathway to decrease sensitivity, reduced uptake by overexpressed efflux transporters, and altered drug detoxification [1, 3].

To address drug resistance, combinatorial therapy with two or more therapeutic agents has been considered as one of the most successful solutions in the clinic, owing to its advantages including synergistic effect, reduced side effects via lowering the administration dose for each drug, improved therapeutic selectivity, and increased patient compliance by decreasing the dosing duration and increasing the length between treatments [4–6].

Paclitaxel (PTX) is one of the most effective chemotherapeutic agents for treating diverse solid tumors, acting through suppressing microtubule disassembly, thereby preventing cell division [7]. However, the clinic use of PTX is markedly hampered by drug resistance mainly stemmed from decreased drug flow and increased drug efflux mediated by efflux pump [2]. The most commonly reported drug-resistant mechanism includes overexpressed efflux transporters, enhanced DNA repair, upregulation of stress-response proteins and inactivation of apoptosis pathway [2, 8, 9]. Besides, increasing evidence demonstrated that autophagy, the mechanism by which cellular material is delivered to the lysosomes for digestion through autophagosomes and consequently offering energy and macromolecular precursors for the cellular metabolism and cell proliferation [10], is closely related with the drug resistance and tumor metastasis as well [11]. In particular, autophagy is able to induce cancer cells’ resistance to the commonly used chemotherapeutic agent, PTX, in breast cancer [12]. Accordingly, autophagy is being considered as a critical factor in inducing PTX’s resistance in cancer therapy [11, 13]. Numerous small-molecular inhibitors of autophagy have been reported to assist the chemotherapeutic agent to kill the stress-tolerant cancer cells efficiently. However, the small-molecular inhibitors lack targeting ability with poor prediction in individual variation [14], and therefore, their high dose of administration is needed, leading to toxic concerns. In contrast, biological drugs, such as protein, peptide, enzyme and gene, are highly attractive in disease treatment, owing to their high potency and high selectivity of action, along with extremely low administration dose (< nM) [15]. Increasing evidence demonstrated that MicroRNAs (miRs), a class of short non-coding RNAs that post-transcriptionally regulate gene expression, are potent regulators of autophagy [16]. And in particular, miR regulators are able to target the autophagy pathway at several different stages with higher efficacy over the small-molecular inhibitors [16]. miR-101 can significantly suppress the autophagy induced by etoposide and rapamycin through knockdown of three genes, STMN1, RAB5A and ATG4D [17]. Moreover, via autophagy inhibition, miR-101 sensitized cancer cells to the chemotherapeutic drugs, 4-hydroxytamoxifen and cisplatin [17, 18]. Nevertheless, it is unknown whether the miR-101 could reverse the drug resistance of PTX via autophagy inhibition and, as a result, improve PTX’s anti-tumor efficacy.

**Scheme 1:**
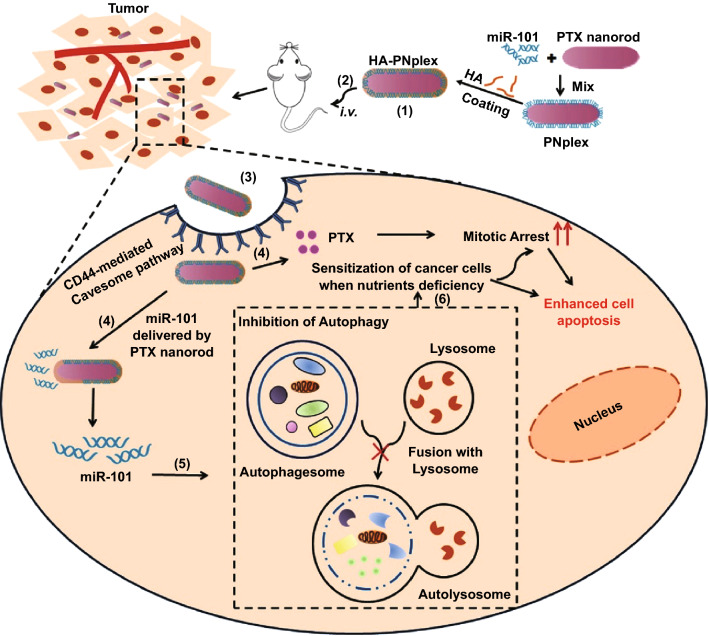
Design and proposed an active mechanism of autophagy inhibition and self-sensitization. (1) Preparation of HA-coated miR-101/PNs complexes (HA-PNplex): miR-101 is condensed on the PNs, followed by HA coating. (2) HA-PNplex is administered via intravenous injection (*i.v.*), accumulate in the tumor site, and (3) enter cancer cells through CD44-receptor-mediated caveolar endocytosis. (4) HA-PNplex release miR-101 and PTX, with miR-101 suppressing the autophagy (5) and, in turn, sensitizing the cancer cells (6) to the anti-tumor carriers, PNs

In our previous reports, we established a drug-delivering-drug (DDD) platform based on drug nanocrystals [19–21]. Via bypassing the digestive endo-lysosomes, the DDD platform maximizes intracellular delivery of biopharmaceuticals and enables the delivery of additional drugs to the target site as well. Here, with the aim of overcoming drug resistance, we propose the co-delivery of the nucleic acid and PTX to cancer cells via a carrier-free strategy (DDD platform, as shown in Scheme 1). Rod-like nanocrystals of the chemotherapeutic agent (PTX), herein referred as PTX nanorods (PNs), are employed as a scaffold for efficient intracellular delivery of miR-101 via a non-lysosomal pathway; and in turn, the delivered miR-101 sensitizes the cancer cells to the cytotoxic PNs by inhibiting the autophagy, ultimately achieving synergistic treatment of cancer. The coating of HA was to shield the positive charge of PNplex and target the CD44 receptors expressed on cancer cells.

## Experimental Section

### Materials

Beta-lactoglobulin (β-LG, No. L3908, 90% purity), fluorescein isothiocyanate isomer I (FITC, 98% purity), rhodamine B isothiocyanate (RITC, 98% purity), IR783 (90% purity), 3-(4,5-dimethylthiazol-2yl)-2,5-diphenyltetrazoliumbromide (MTT, 98% purity) and polyethylenimine (PEI, 408727, 25 kDa) were purchased from Sigma-Aldrich Co., Ltd. (St. Louis, MO, USA). PTX (99% purity) was obtained from Yew Biotechnology Co., Ltd. (Jiangsu, China). The dye, P2 (*λ*_abs_/*λ*_em_ = 708 nm/732 nm), was a gift from Prof. Wei Wu (School of Pharmacy Fudan University, Shanghai, China) [22, 23]. Taxol (marketed product of PTX) was from Bristol-Myers Squibb (China) Investment Co., Ltd. (Shanghai, China). Human microRNA-101 (miR-101) mimics and Cy5-miR-101 mimics were purchased from Genepharma Co., Ltd. (Shanghai, China). Hyaluronic acid (HA) was obtained from Shandong Furuida Pharmaceutical Co., Ltd. (6600 Da, Shandong, China). Nystatin and methyl-β-cyclodextrin (M-CD) were acquired from Aladdin Co., Ltd. (Shanghai, China). Caveolae Marker (Alexa Fluor^®^ 488, ab185043), primary antibody for p62 (ab109012) and LC3 (ab48394), GADPH (ab37168) and HRP-conjugated secondary antibody (ab205718) were purchased from Abcam Trading Co., Ltd. (Shanghai, China). Alexa Fluor^®^ 488-cholera toxin subunit B (CTB), Pierce™ BCA Protein Assay Kit, fetal bovine serum (FBS), RPMI-1640, Dulbecco’s Modified Eagle Medium (DMEM), lyso-tracker green or red, trypsin miScript II Reverse Transcription Kit and miScript SYBR Green PCR kit were from Thermo Fisher Scientific (Waltham, MA, USA). FITC phalloidine was from Shanghai Yeasen Biotechnology Co., Ltd. (Shanghai, China). Bafilomycin A_1_ (BFA) was purchased from Dalian Meilun Biotech Co., Ltd. (Dalian, China). The In Situ Cell Death Detection Kit was purchased from F. Hoffmann-La Roche Ltd. (Basel, Switzerland). Annexin V-FITC/PI, blocking buffer and Hematoxylin & Eosin Staining (H&E staining) kits were obtained from the Beyotime Institute of Biotechnology (Haimen, China). RNA isolation kit was purchased from QIAGEN (Hilden, Germany). SDS-PAGE gel and ECL Detection Kit were from Bio-Rad (Hercules, CA, USA).

### Cell Cultures

The cell lines, MCF-7 (human breast adenocarcinoma), Caco-2 (human colon colorectal adenocarcinoma) and 4T1 (murine mammary carcinoma), were purchased from Nanjing KeyGEN Biotech Co., Ltd. (Nanjing, China). mRFP/EGFP-LC3-MCF-7 cell line was purchased from Shanghai Sciencelight Biology Science and Technology Co., Ltd. MCF-7 and mRFP/EGFP-LC3-MCF-7 cell lines were maintained in DMEM medium supplemented with 10% FBS and 1% penicillin/streptomycin at 37 °C, 5% CO_2_ and 100% humidity and were split when confluent. Caco-2 cells were cultured in the same conditions, except for being kept in 20% FBS. 4T1 cells were cultured in the same conditions, except for being kept in RPMI 1640 medium.

### Preparation and Characterization of Nanoparticles

Cationic β-LG (CLG) was prepared as described in previous reports [19, 21]. PNs were prepared via an antisolvent precipitation. Briefly, 1 mL acetone with 20 mg of PTX (organic phase) was added to 10 mL of aqueous phase with stabilizer, CLG (10 mg), under vigorous stirring in an ice-water bath (4 °C), followed by ultrasonic treatment using an ultrasonic probe (20–25 kHz, Scientz Biotechnology Co., Ltd., Ningbo, China) at 400 W for 15 min and centrifugation at 10,000 *g* for 10 min. PNs/miR-101 complex (PNplex) was first prepared via mixing miR-101 with PNs at equal volume with gentle vortexing for 30 s and further incubation at room temperature (RT) for 30 min. After that, the PNplex was incubated with HA solution at concentrations of 0–2 µg mL^−1^ to form HA-coated PNplex (HA-PNplex).

The dye-labeled nanoparticles were prepared by a similar procedure. For FITC-, aza-BODIPY(P2)- or IR783-labeled nanoparticles, the dye and PTX were dissolved in the organic phase together prior the addition into the aqueous phase. For Cy5-labeled nanoparticles, Cy5-miR-101 rather than miR-101 was used to prepare the nanoparticles. Dual-labeled nanoparticles were prepared by condensing Cy5-miR-101 on FITC-PNs followed by the coating with HA.

The particle size and zeta potential of the nanoparticles were measured three times with a 90Plus Particle Size Analyzer (Brookhaven Instruments, Holtsville, NY) at 25 °C according to a dynamic light scattering (DLS) principle. The morphology of nanoparticles was examined using a JEM-1230 transmission electron microscope (TEM, Tokyo, Japan). The prepared nanoparticles with 200-fold dilution were placed on a copper mesh TEM grid for 10 min and then the excess of liquid removed with a filter paper followed by the addition of one drop of 2% (w/w) phosphotungstic acid for 1 min to stain the nanoparticles. Then, the copper mesh grid was dried at 25 °C for 15 min after the removal of the phosphotungstic acid excess.

The binding of miR-101 to the nanoparticles was determined by agarose gel electrophoresis. The gels were prepared with 0.8% agarose in Tris buffer containing ethylenediaminetetraacetic acid (EDTA) solution (pH 8.0). After mixing the prepared PNplex with GelRed (Generay Biotechnology, China) following the manufacturer’s protocol, the gel electrophoresis was conducted at 110 V for 30 min. The gel was then imaged using a Bio-Rad high-sensitivity chemiluminescence imaging system (Chemidoc XRS+, USA).

The stability of nanoparticles was evaluated by incubating them in culture medium with 10% of serum at 37 °C for 4 days and measuring their size by DSL as described above.

### Flow Cytometry

To assess the endocytic mechanism, the cells cultured in 12-well plates at a density of 2 × 10^5^ cells/well for 48 h were pretreated with uptake inhibitors, nystatin (10 mM) and M-CD (2.5 mM), for 30 min and then incubated with FITC or Cy5 labeled nanoparticles in serum-free media at 37 °C for 4 h, washed three times with PBS and detached by trypsinization and finally subjected to fluorescence determination by flow cytometry (BD FACSCalibur, USA).

To assess the autophagy flux, the mRFP and EGFP fluorescence were quantified. MCF-7 cells stably expressing mRFP/EGFP-LC3 were seeded on 12-well plates and incubated with different formulations for 6 h, followed by treatment with BFA (10 nM) for 30 min. Then, the cells were trypsinized to measure the fluorescence by flow cytometry.

For the intracellular fate study, MCF-7 cells (2 × 10^5^ cells/well) were first incubated with aza-BODIPY-labeled HA-PNplex in serum-free media at 37 °C for 3 h and then rinsed with PBS and cultured in serum-free medium at 37 °C for additional 0, 1, 3 and 7 h. After that, cells were washed, trypsinized and analyzed by flow cytometry.

### Confocal Microscopy Imaging

For assessing co-localization within intracellular compartments, Caco-2 cells were incubated with dye-labeled nanoparticles in DMEM for 4 h at 37 °C, followed by staining with caveolae makers, Alexa Fluor^®^ 488-Cave-1/F-actin/CTB for 3 h, or lyso-tracker red or green for 1 h. Then, the cells were rinsed with PBS and observed under an LSM700 confocal laser scanning microscopy (CLSM, Carl Zeiss, Germany). The co-delivery of PNs and miR-101 to cells was studied by incubation of dual-labeled nanoparticles in Caco-2 cells at 37 °C for 3 h.

For the evaluation of autophagy fluxes, MCF-7 cells expressing mRFP/EGFP-LC3 were treated with different formulations for 6 h, washed with PBS three times, fixed in the 4% paraformaldehyde for 10 min and observed in the LSM700 CLSM. To investigate the intracellular fate, MCF-7 cells pre-incubated with aza-BODIPY labeled nanoparticles were fixed and observed under the LSM700 CLSM after stained with DAPI.

### Polymerase Chain Reaction (PCR)

MCF-7 cells cultured in 6-well plates at a density of 2 × 10^5^ cells/well for 48 h were transfected with different formulations for 24 h and subjected to extraction of total RNA using RNA isolation kit (Qiagen, Germany). Reverse transcription reaction of miRNA and mRNA to cDNA was performed using miScript II Reverse Transcription Kit (Qiagen, Germany) and Taqman^®^ Reverse Transcription Reagents (Thermo Fisher, United States), respectively. MiR-101 and mRNA levels of LC3II and p62 were quantitatively assayed in a real-time PCR system (Eppendorf, Germany) using miScript SYBR Green PCR kit. miR-101 and mRNA were normalized to U6 snRNA and GADPH levels, respectively. Primers were designed as: miR-101: 5′-UACAGUACUGUGAUAACUGAA-3′, LC3II forward: 5′-ATGTTGGTTAGTGGCAGAAGAG-3′, LC3II reverse: 5′-ACAGGGTATCATTCACAAAGTC-3′, p62 forward: 5′-CACCTCCTCCACCACCTGTTCC-3′, p62 reverse 5′-GCCCGCTGTCCGTGCTCTTG-3′, GPADH forward: 5′-CATCAAGAAGGTGGTGAAGCAGG-3′, GPADH reverse: 5′-AAAGGTGGAGGAGTGGGTGTCG-3′.

### Western Blot (WB)

MCF-7 cells in 6-well plates at a density of 2 × 10^5^ cells/well were incubated with formulations for 24 h, followed by protein isolation with radioimmunoprecipitation assay buffer and determination with a BCA protein assay kit. After that, the cell lysates were mixed with loading buffer, boiled at 100 °C for 5 min, loaded on the wells of SDS-PAGE gel, transferred by electroporation to PVDF membrane, incubated with blocking buffer for 1 h at RT, incubated with anti-p62 and anti-LC3 II overnight at 4 °C and HRP-conjugated secondary antibody at RT for 1 h, and stained with an ECL chemiluminescence kit (KeyGEN Biotech., China), respectively. GADPH was used as the loading control. Bands were quantified by an ImageJ software (National Institutes of Health), and protein levels were normalized to GADPH.

### Cell Migration Assay

Cell migration assay was performed using Transwell^®^ chambers (Corning) in 4T1 and MCF-7 cell lines. A cell suspension (200 µL) at a density of 6 × 10^5^ cells mL^−1^ was transferred into the upper chamber of the Transwell^®^ and incubated for 24 h while 1 mL of RPMI 1640 or DMEM with 20% FBS was added to the lower Transwell^®^ chamber to act as chemoattractants. Twenty-four hours later, the cells that did not migrate through the pores of the filter were carefully removed with a cotton-tipped swab. The filters were fixed in 4% paraformaldehyde, dried at RT and stained with crystal violet. They were then observed under a light microscope. The optical density (OD) was measured in a microplate reader at 570 nm after crystal violet was dissolved in 33% (w/v) acetic acid.

### In Vitro Cytotoxicity

Cells seeded in 96-well plates at a density of 5 × 10^3^ cells/well were first cultured for 48 h and then incubated with drug formulations for 24 h in MCF-7 cells and 4 h in Caco-2 cells. Cell viability was assessed by the standard MTT assay.

MCF-7 cells were cultured first in 12-well plates at a density of 5 × 10^3^ cells/well for 48 h and subsequently incubated with drug formulations for 24 h, detached, washed with PBS three times, and stained with Annexin V-FITC/PI apoptosis kit according to the manufacturer’s protocol. The apoptosis rates were calculated using flow cytometry.

### In Vivo Imaging and Biodistribution

The animals used in all experiments received care in compliance with the Principles of Laboratory Animal Care and the Guide for the Care and Use of Laboratory Animals. Animal experiments followed a protocol approved by the China Pharmaceutical University Institutional Animal Care and Use Committee. The female Balb/C nude mice (5-week old, 18–22 g) were purchased from the College of Veterinary Medicine Yangzhou University (License No: SCXK (Su) 2012-0004, Yangzhou, China).

The MCF-7 tumor-bearing Balb/C nude mice with a tumor volume of 500 mm^3^ were dosed with IR783-labeled nanoparticles via tail vein at a single IR783 dose of 2.5 mg kg^−1^, according to the animal’s weight. To localize the dye-labeled nanoparticles, the mice were observed in an imaging system (IVIS Spectrum, PerkinElmer, USA) at an excitation wavelength of 768 nm and an emission wavelength of 789 nm, at predetermined time points. After in vivo imaging, the euthanized mice were killed to collect the major organs for *ex vivo* imaging.

### In Vivo Anti-Tumor Activity

The MCF-7 tumor-bearing Balb/C nude mice were divided into nine groups (*n* = 6). Formulations were administered via tail vein with PTX 10 mg kg^−1^ and miR-101 1 mg kg^−1^ every 3 days in a total of 6 injections, according to the animal’s weight. Tumor volumes were measured every time before injection. At the third day after the final administration, the animals were killed to collect the tumors for H&E staining, immunohistochemical and WB analysis.

### Statistical Analysis

One-way analysis of variance was performed to assess the statistical significance of the differences between samples. The results are expressed as the mean ± standard deviation (SD). *P* < 0.05 indicated significant differences.

## Results

### Structure Characterization of Nanoparticles

**Fig. 1:**
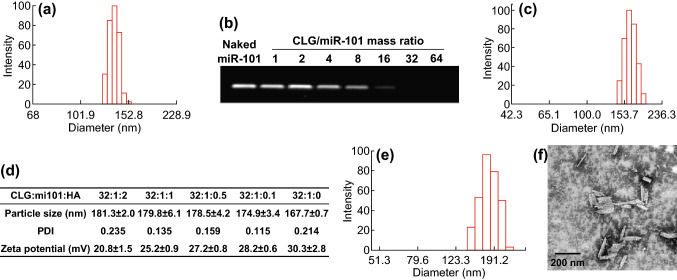
Preparation and characterization. Size distributions of **a** PNs and **c** PNplex. **b** Gel electrophoresis of PNplex with different mass ratios of stabilizer (CLG)/miR-101. **d** Particle size, PDI and zeta potential (*ξ*) of HA-PNplex with different mass ratios of CLG/miR-101/HA. **e** Size distribution and **f** TEM image of HA-PNplex from the optimized formulation

The DDD platform for the co-delivery was prepared by a process that assembled miR-101 on 140-nm PNs (Fig. 1a) via electrostatic interaction between the negatively charged microRNA and positively charged PTX nanorods (PNs, *ξ* = 35.3 mV) followed by a coating of hyaluronic acid (HA) (see Scheme 1). The formulation of PNs/miR-101 complexes (PNplex) was first optimized by combining different mass ratios of stabilizer/miR-101. Gel electrophoresis assay displayed no bands when the mass ratio was ≥ 16 (Fig. 1b), indicating the gene was compressed well. The PNplex with the mass ratio of 16 showed a size of 167.7 nm and zeta potential of 30.3 mV (Fig. 1c). This PNplex formulation was then selected for HA coating to target the CD44 receptors expressed on cancer cells [24] and shield the positive charge. The HA coating increased the size of PNplex from 167.7 to 181.3 nm along with charge reduction from 30.3 to 20.8 mV with an increasing amount of HA in the coating (Fig. 1d). The HA-coated PNplex (HA-PNplex) with a size of 181.3 nm was chosen for subsequent studies considering their lowest surface charge among all formulations. Under TEM observation, the HA-PNplex exhibits a rod-like morphology with a size of 170–200 nm in length (Fig. 1f), being in line with the DLS results (Fig. 1e). The HA-PNplex possessed total drug loading up to 67.27% (drug mass compared to total weight of dried HA-PNplex), 66.24% for PTX and 1.03% for miR-101, exhibiting significant superiority over the conventional nanomedicine with drug loading of less than 10%. Furthermore, their size was not significantly altered after incubation in 10% serum at 37 °C for 4 days, indicating prolonged stability and superior resistance against serum-mediated aggregation and degradation (Fig. S1).

### Cytosol Delivery, Intracellular Fate and Efficient Gene Delivery

The internalization pathway of the developed nanoparticles was investigated using two cell lines, MCF-7 and Caco-2 with high expression of CD44 receptors [25, 26]. The cellular uptake of dye-labeled nanoparticles was significantly suppressed by the inhibitors, nystatin and M-CD (Fig. S2a, b). These two inhibitors are known to block the cholesterol-dependent internalization pathway [27], being closely linked with the caveolar endocytosis that enables materials to enter the cells bypassing the digestive lysosomes [19, 28]. To confirm the caveolar internalization, the uptake was studied in Caco-2 cells whose surface area of 50% is occupied by caveolae [29]. Profound inhibition was observed (Fig. S2b), demonstrating the caveolae-dependent internalization. Observation with CLSM demonstrated that the Cy5-nanoparticles co-localized well with the Cave-1 and two caveolar transporters, cholera CTB and F-actin (Fig. S2c–e). The nanoparticles showed minor co-localization with the lysosomes after 3-h incubation (Fig. S3**)**. Additionally, the caveolar endocytosis was time-dependent in a 4-h period (Fig. S4a, b). The dual-labeled nanoparticles demonstrated the signals of FITC-labeled PNs (FITC-PNs) and Cy5-miR-101 simultaneously by flow cytometry assay and showed yellow fluorescence in the confocal images (Fig. S4c, d), confirming the co-delivery of the two drugs.

Unlike other nanoparticles that would release their payloads rapidly after internalization due to digestion in the lysosomes, the drug nanorods have to take time to be dissolved for drug release. Accordingly, we explored the potential intracellular fate of HA-PNplex by incorporating an aza-BODIPY dye into the PNs. Aza-BODIPY was used as this dye can emit red fluorescence upon exposure and aggregation in a lipid environment [22]. Intracellular fate study showed that the internalized nanoparticles began to disintegrate 3 + 1 h, but was more evident at 3 + 3 h and in particular at 3 + 7 h (Fig. S5), an indicator that the nanoparticles released the two payloads significantly after entering into the cells. All together, these results suggest that the nanoparticles obtained cellular entry through a caveolae-mediated process without being captured by the lysosomes and taking approximately 7 h to release the two drugs.

**Fig. 2:**
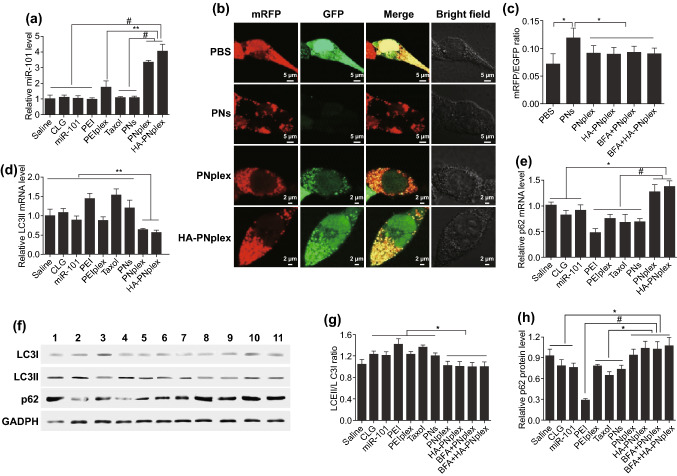
Intracellular delivery of miR-101 and in vitro inhibition of autophagic flux. **a** miR-101 expression in MCF-7 cells with PTX concentration of 10 μg mL^−1^ or a miR-101 at concentration of 100 nM after 24 h incubation at 37 °C as determined using PCR assay (*n* = 3, ^#^*P* < 0.001 and ^**^P < 0.01). **b**–**h** In vitro inhibition of autophagic flux. **b** CLSM images showing the autophagy flux in mRFP/EGFP-MCF-7 cells with PTX concentration of 10 μg mL^−1^ or a miR-101 at concentration of 100 nM after 24 h incubation at 37 °C. The green fluorescence is negatively correlated with the fusion of autophagosomes and lysosomes. The yellow fluorescence indicates the absent fusion of autophagosomes and lysosomes. **c** Quantitative mRFP/EGFP ratio determined by flow cytometry (*n* = 3, ^*^P < 0.05). **d** LC3II and **e** p62 mRNA expression level in MCF-7 cells assessed using PCR assay (*n* = 3, ^*^P < 0.05, ^**^P < 0.01 and ^#^*P* < 0.001). **f** LC3I, LC3II and p62 expression level in MCF-7 cells determined using the WB assay. Quantitative analysis of **g** LC3II/LC3I ratio and **h** p62 based on WB assay (*n* = 3, ^#^*P* < 0.001 and ^*^P < 0.05). The internal control for normalizing protein expression was GADPH. Formulations: 1, saline; 2, CLG; 3, naked miR-101; 4, PEI; 5, PEI/miR-101 complexes (PEIplex); 6, Taxol; 7, PNs; 8, PNplex; 9, HA-PNplex; 10, BFA+PNplex; 11, BFA+ HA-PNplex

Conventional nanoparticles for gene delivery would be trapped within the lysosomes post-internalization, resulting in less than 2% of gene drug escaping to the cytosol and significantly compromising the transfection efficacy [30, 31]. Consequently, the present nanoparticles had potential to improve the intracellular delivery of miR-101. As expected, PCR results revealed approximately a two- and threefold increase in miR-101 level in MCF-7 cells after 24-h incubation with PNplex and HA-PNplex (Fig. 2a), compared to the commonly used PEI carrier.

### In Vitro Inhibition of Autophagic Flux

Regarding the autophagy, microtubule-associated protein 1A/1B-light chain 3 (LC3) is a robust marker of autophagosomes [32]. Here, we first utilized MCF-7 cells expressing mRFP/EGFP-LC3 reporters to assess the autophagy, in which autophagosomes have both mRFP and EGFP signals. However, the autolysosomes display mRFP signal only when the signal of EGFP is quenched in the acidic lysosomal environment [33, 34]. The PBS-treated cells emitted both signals of mRFP and EGFP and demonstrated the little fusion of autophago- and lysosomes (Figs. 2b and S6). In contrast, the treatment with PTX formulation, PNs, noticeably quenched the EGFP signal (Fig. 2b, c), indicating the fusion of autophago- and lysosomes and the resultant triggering of autophagy. Importantly, significant mRFP and EGFP signals were exhibited after treatment with the miRNA-101 formulations, PNplex and HA-PNplex, along with the lower mRFP/EGFP ratio compared to cells treated with PNs (Fig. 2c). Moreover, the ratio was not changed after additional treatment with BFA, a late phase autophagy inhibitor that prevents autophagosome–lysosome fusion and LC3II degradation, and therefore indicating the autophagy inhibition was caused by the suppression of autophagic flux **(**Fig. 2c**)**. In addition, in the process of autophagy, a cytosolic form of LC3 (LC3I) is conjugated to phosphatidylethanolamine (PE), subsequently forming LC3-PE (LC3II) and recruited to autophagosomal membranes. Also, p62 is selectively degraded by autophagy and serves as a marker of autophagy induction [32, 35]. Therefore, we further confirmed the autophagy by assessing the levels of LC3 and p62. As depicted in Fig. 2d, e, PNplex and HA-PNplex enabled 40% and 50% reduction in LC3II mRNA and an increase in the mRNA expression of p62 by 25% and 30%, respectively. WB analysis confirmed these results at the protein level **(**Fig. 2f**)**. Quantification of WB results revealed that the ratios of LC3II/I and p62 protein levels for these two nanoparticles were similar to that of the saline group (Fig. 2g, h). And again, additional treatment with BFA after administration of these two nanoparticles had little influence on the ratios and p62, implying the inhibition of autophagic flux. Taken together, the PNplex and HA-PNplex inhibited the autophagic flux markedly, whereas the administration of PTX formulations, Taxol and PNs induced autophagy.

### Enhanced Cytotoxicity, Synergistic Effect and Anti-Metastasis

**Fig. 3:**
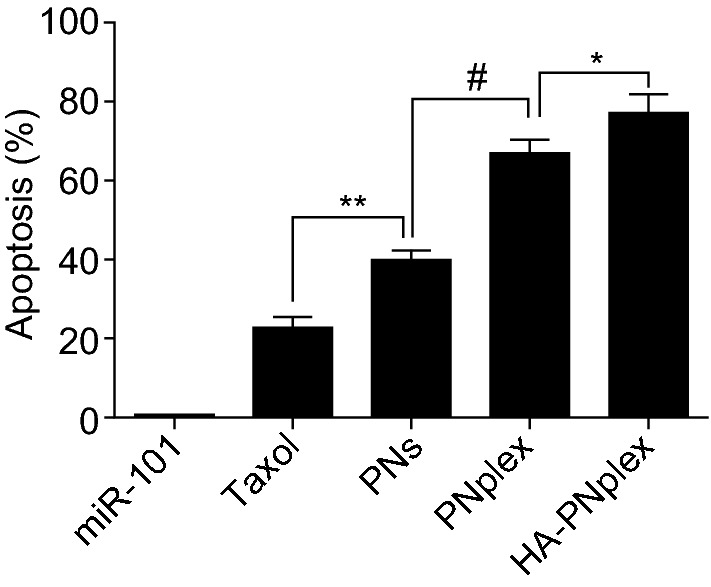
In vitro apoptosis. Percentage of cell apoptosis in MCF-7 cells induced by diverse formulations after 48 h incubation at a fixed PTX concentration of 10 μg mL^−1^ or miR-101 at 100 nM at 37 °C as determined by flow cytometry (*n* = 3, ^**^P < 0.05, ^**^P < 0.01, and ^#^*P* < 0.001)

Autophagy is intimately related to the cytotoxicity, drug resistance and metastasis in chemotherapy [36]. Here, we first examined the cytotoxicity. Naked miR-101 showed little toxicity to the cancer cells at the studied concentrations (Fig. S7a, b). Compared with PNs, PNplex and HA-PNplex killed the cancer cells with higher efficiency at PTX concentrations of 1–100 µg mL^−1^ (Fig. S7c, d). Flow cytometry assay indicated the apoptosis induced by HA-PNplex and PNplex was roughly 77% and 67% (Figs. 3 and S7e), approximately 2- and 1.7-fold as much as that of PNs or 3.4- and 2.9-fold as great as that from Taxol (free PTX formulation). These results implied that formulating miR-101 into PNs was able to significantly promote the cytotoxicity of the chemotherapeutic agent to the cancer cells.

Then, the migration of 4T1 and MCF-7 cells was studied in a transwell chamber system (Fig. S8a). The HA-PNplex and PNplex blocked the cell migration significantly (Fig. S8b-e) and the HA-PNplex, in particular, reduced the migration by > two- and > threefold for 4T1 and MCF-7 cells, respectively, in contrast with PNs.

To study the potential synergistic effect between PTX and miR-10, we calculated the combination index (*CI*) based on the cytotoxicity induced by HA-PNplex with two ratios of PTX/miR-101 (16:1 and 32:1), which *Cl *> 1, *C l *= 1, and *Cl *< 1, represent synergism, additive effect and antagonism, respectively. As depicted in Fig. S9, at inhibition rate (*F*_a_) of > 20%, the *Cl* values from the two ratios were less than 1 and, as a result, indicated the synergism between them.

### Tumor Targeting, In Vivo Anti-Tumor Activity and Biocompatibility

Biodistribution and tumor targeting in vivo were first studied in MCF-7 tumor-bearing mice after the injection of free IR783 or IR783-labeled nanoparticles (Figs. S10 and S11). The IR783-nanoparticles accumulated in the tumor efficiently after 2-h or even 24-h administration compared to the free IR783 and the HA coating further promoted the accumulation due to specific binding to CD44 receptors on cancer cells [24]. These results demonstrated that the HA-PNplex and PNplex possessed significant tumor-targeting ability.

**Fig. 4:**
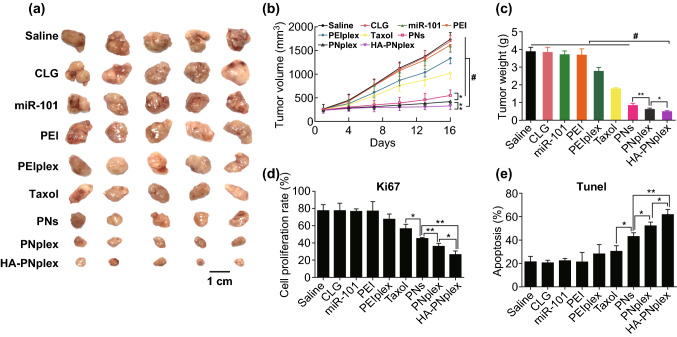
Anti-tumor efficiency. Different formulations (0.2 mL) were injected into MCF-7 tumor-bearing nude Balb/C mice via tail vein every 3 days at a PTX dose of 10 mg kg^−1^ and miR-101 dose of 1 mg kg^−1^, according to the animal’s body weight. On day 16 at the end of the experiment, the tumor tissues were collected for further examination. **a** Representative images of the tumors collected from mice at the end of the experiment. **b** Tumor growth curves (*n* = 6, ^*^P < 0.05, ^**^P < 0.01 and ^#^*P* < 0.001). **c** Tumor weight after treatment with the various formulations (*n* = 6, ^*^P < 0.05, ^**^P < 0.01 and ^#^*P* < 0.001). Quantitative analysis of **d** cell proliferation and **e** apoptosis (*n* = 3, ^*^P < 0.05 and ^**^P < 0.01)

Second, the anti-tumor efficacy in MCF-7 tumor-bearing mice was investigated post-injection of different formulations (Fig. 4). In contrast with the formulation of PTX alone, the combinatorial formulation, HA-PNplex, inhibited the tumor growth markedly, displaying an almost zerofold increase in the tumor volume (Fig. 4a–c). The PNplex formulation also exhibited significantly improved suppression of tumor growth. Further Ki67 and Tunel examinations on the isolated tumor collected at the end of treatment indicated increased rates of anti-proliferation and apoptosis by HA-PNplex, approximately 100% and 50%, respectively, compared with PNs (Fig. S13). The induced apoptosis in the tumor by the combined formulation was confirmed by H&E staining (Fig. S13).

Additionally, safety examination on the major tissues sampled at the end of treatment revealed that HA-PNplex and PNplex did not cause toxicity to these tissues, whereas the classic gene carrier, PEI, its formulation PEIplex, and Taxol-induced liver toxicity (Fig. S14).

### In Vivo Gene Delivery and Inhibition of Autophagy

**Fig. 5:**
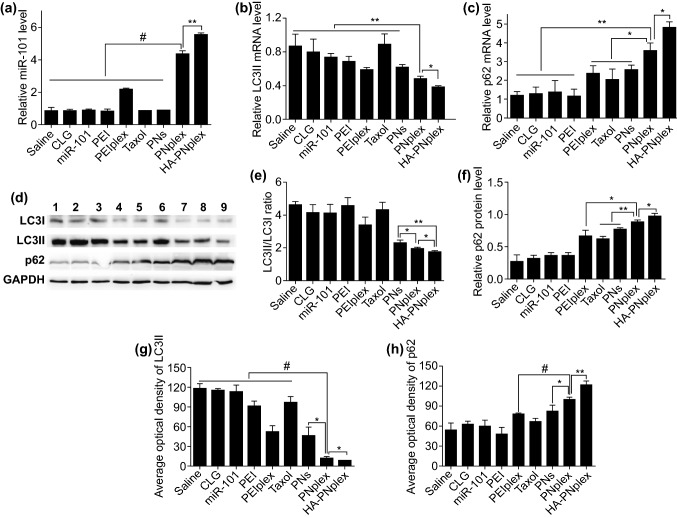
In vivo miR-101 expression and inhibition of autophagy flux. **a** miR-101 expression level in tumor was determined by PCR assay (*n* = 3, ^**^P < 0.01 and ^#^*P* < 0.001). The tumors were collected from mice on day 16 at the end of the experiment. **b**–**h** In vivo inhibition of autophagy flux. At the end of the experiment for antitumor efficiency, the tumor tissues were isolated. **b** LC3II and **c** p62 mRNA expression level in tumor determined by PCR assay (*n* = 3, ^*^P < 0.05 and ^**^P < 0.01). **d** LC3I, LC3II and p62 expression level in tumor determined using the WB assay. Formulations: 1, saline; 2, CLG; 3, naked miR-101; 4, PEI; 5, PEI/plex; 6, Taxol; 7, PNs; 8, PNplex; 9, HA-PNplex. The internal control for normalizing protein expression was GADPH. Quantitative analysis of **e** LC3II/I ratio and **f** p62 in tumor (*n* = 3, ^**^P < 0.01 and ^*^P < 0.05). The immunohistochemistry quantification of **g** LC3II and **h** p62 in the tumors (*n* = 3, ^*^P < 0.05, ^**^P < 0.01 and ^#^*P* < 0.001)

First, we examined the miR-101 level in the tumor at the end of treatment by PCR assay. As depicted in Fig. 5a, HA-PNplex and PNplex had an approximately three- and twofold increase in the miR-101 level over the PEIplex formulation. These findings correlate well with the in vitro results (Fig. 2a) and indicate the potential of PNs for intracellular gene delivery. Next, we assayed the in vivo inhibition of autophagy in the tumor after treatment regarding the mRNA expression and protein levels of LC3, LC3II/I ratio and p62. HA-PNplex allowed for 2.3-fold reduction in LC3II mRNA and 4.8-fold increase in p62 mRNA over the saline control, while enabling approximately a 0.5-fold decrease in LC3II mRNA and twofold increase in p62 mRNA compared with PEIplex control (Fig. 5b, c). WB assay demonstrated that treatment with HA-PNplex reduced the LC3II/I ratio and p62 protein level significantly over the treatment with saline or PEIplex (Fig. 5d–f). Immunohistochemical analysis for the collected tumor tissues revealed marked positive staining for tumor treated with HA-PNplex (Fig. S15). This treatment promoted a decrease in the optical density of LC3II by > 10-fold and an increase for p62 by > twofold over the saline control (Fig. 5g, h). These results demonstrated that HA-PNplex noticeably inhibited the autophagic flux in vivo, consistent with the autophagy inhibition seen in vitro (Fig. 2).

## Discussion

There are three approaches for combined treatments [4]: (1) co-dosing of conventional formulations, (2) co-dosing of two or more nanomedicines [37] and (3) co-delivering multiple drugs via loading them in a nanoparticle system [38]. Of these strategies, nanoparticle-mediated co-delivery has unique merits to overcome drug resistance by providing more precise control over the spatiotemporal release of each drug and the delivery of approximate drug ratio to the target site [39, 40]. Nonetheless, conventional nanoparticles have low drug-loading capacity (< 10%, w/w) thus limiting their use in long-term therapies [20, 41]. This low loading capacity becomes more limiting in co-delivery approaches, as multiple drugs need to be loaded. Furthermore, nanoparticles are typically arrested in the digestive lysosomes [30, 31], significantly hindering the drug release in the cytosol. This is particularly crucial when delivering nucleic acids that require reaching the cytosol in considerable amounts in order to exert its biological activity. In this study, by using the drug nanorods, rather than polymeric nanoparticles as vectors, the DDD platform, HA-PNplex, was prepared to co-deliver PTX and miR-101 via a simple mixing process and with a total drug loading up to 67.27%, sixfold greater than the conventional nanoparticle-based co-delivery systems with < 10% payload. We believe that, in drug-loading capacity, the developed nanoplatform has overwhelmed advantages than the reported nanoparticles. More importantly, the HA-PNplex specifically delivered miR-101 to cancer cells without being arrested within the lysosomes, offering significant merits over other nanoparticles with < 2% lysosomal escape of nucleic acid to the cytoplasma after internalization [30, 42, 43].

Combinatorial therapy based on nanoparticle-mediated co-delivery holds promise to tackle the problem of drug resistance and to improve the drug delivery. However, only few cases of clinical translation have been successful, despite numerous publications. Until now, only one nanoparticle-based product for combined therapy, VYXEOS (liposomal daunorubicin and cytarabine), has been approved. Undoubtedly, complicated preparation is a dominant obstacle for the translation. Here, the preparation of nanocrystal-based combinatorial therapy is extremely simple, just needing to mix the nucleic acid and the nanocrystals together. Furthermore, the developed DDD is universally adopted to overcome drug resistance and to achieve nanoparticle-mediated combinatorial therapy. We may utilize another type of drug nanocrystal as carriers to deliver second biologic macromolecules even if the intracellular-delivery efficacy is not as great as that of the present platform. Additionally, the platform can be modified easily with ligands for active targeting to further improve the drug delivery. In this study, the HA coating targeting CD44 receptors allowed for enhanced delivery, exhibiting as 1.3-fold increased uptake at 4 h after incubation (Fig. S4) and 1.6-fold increased accumulation in the tumor site at 2 h post-injection (Fig. S11) and, as a result, enabled improved cytotoxicity and anti-migration in vitro and in vivo. Besides, the ligand decoration reduced the positive charge of the DDD platform (Fig. 1d) and benefited the improvement of blood circulation and, subsequently, improved accumulation and penetration in tissues. As shown in Fig. S10c, d, the accumulation in the tumor, liver, lung and kidneys of HA-PNplex at 24 h post-injection was higher than that of PNplex. In particular, compared with PNplex, HA-PNplex had significantly higher lung accumulation. The increased accumulation might be related to the reduced positive potential from 30.3 to 20.8 mV due to the HA coating. Previous reports demonstrated that nanoparticles with high positive potential tended to associate with lung epithelial cells, and whereas, the charge-reduced nanoparticles would concentrate within interstitial spaces and then combat against the clearing by alveolar macrophages [44]. On the other hand, larger particles (1–3 µm) prefer to deposit in the lung [45, 46]. The reduced potential would potentially result in the aggregation of HA-PNplex in vivo and consequently increase the lung accumulation of the nanoparticles. Additional experiments are needed to explore the mechanism. Overall, the platform represents a robust strategy for combined treatment and its clinical translation is ongoing.

Via cytosol delivery of nucleic acid with autophagy activity using PTX nanorods, the PTX’s anti-tumor efficacy was enhanced greatly, along with two- to threefold increase in apoptosis in vitro and in vivo, fivefold reduction in the tumor volume as compared with PTX used alone. These results indicated that autophagy inhibition is efficient to combat against the drug resistance of PTX. The enhancement was ascribed to the effective gene transfection via a non-lysosomal internalization of the present platform and the potential synergy between the two drugs. It is well known that intracellular delivery of gene or protein drugs is challenging due to their rapid degradation in digestive endo-lysosomes [30, 42, 43], despite the diverse types of carriers used. Via bypassing the digestive endo-lysosomes, the developed platform maximized the intracellular delivery of miR-101. Additionally, the system allowed for rapid intracellular release of nucleic acid and sustained release of small-molecular drug over time. Our previous report indicated that the biological protein loaded in the DDD platform was released up to 80% at 2 h and while, the PTX was released in a sustained pattern, along with < 30% at 24 h, demonstrating their non-synchronized release [19]. Indeed, the study of intracellular fate of the DDD revealed delayed release of PTX (Fig. S5). In the combined therapy (nucleic acid and small-molecular drug), the two drugs are spatio-temporarily discrepant in the cytosolic target and the exertion of activity, and especially, > 24 h must be taken for the nucleic acid to exhibit its activity [47]. Therefore, the sequential release of the payloads from nanoparticles is necessary [48, 49]. Disappointingly, the reported co-delivery system tends to release the payloads simultaneously due to its decomposition under the lysosomal conditions, thus compromising the synergistic effect. By contrast, the system developed here could allow the synergistic effect between the two drugs to be maximized. Precise mechanism of the synergy regarding sequential release of the payloads from the DDD platform should be further investigated.

Nanosized cytotoxic drug efficiently discounted the autophagy induced by the drug. Numerous evidence demonstrated that the administration of a chemotherapeutic drug and the drug-loaded nanoparticles induced autophagy to a similar extent [50–52], therefore decreasing the sensitivity of cancer cells to chemotherapy. Moreover, the endocytosis of nanoparticles that causes lysosomal dysfunction and stress response would induce autophagy as well [53, 54], which most of nanoparticles obtain cellular entry through the lysosomal pathway. Conversely, in this study, the administration of PNs significantly suppressed autophagy in vitro and in vivo induced by free PTX (Figs. 2 and 5). Our previous reports indicated that the PNs entered cells via the non-lysosomal pathway [55, 56] and probably affected the fusion of lysosomes with autophagosomes. As a result, we speculate that the nanoparticles that could be uptaken via bypassing the lysosomes are able to resist the autophagy caused by the cytotoxic drug and, in turn, elevate the drug’s activities. The assumption was ascertained by the cytotoxicity examinations (Figs. 3, 4 and S7). This is a first report that the autophagy stemmed from nanoparticles could be reduced potently via non-lysosomal endocytosis. The work offers a unique strategy to combat against autophagy in chemotherapy using nanoparticle drug delivery system.

Autophagy pathway has been considered well to be involved in numerous human diseases from inflammatory disease to cancer, and accordingly, lots of autophagy regulators are developed [14, 57]. However, a question is raised that how to exert the effects of the regulators in disease treatment. Few reports are linked to this issue. Here, we found that by delivering a macromolecular autophagy inhibitor with drug nanorods could in turn elevate the activities of the nanorod themselves significantly. Besides, previous reports demonstrated that the developed DDD platform was able to deliver the second small molecules via formulating them into/on the drug nanorods to overcome the drug resistance [20, 58]. Therefore, the present study offers a promising platform to efficiently utilize autophagy modulators, despite the types of regulators and translate them into the clinic.

## Conclusions

By employing drug nanorods with potent cytotoxicity as a vehicle for intracellular delivery of nucleic acid via a non-lysosomal pathway, a generalized approach for drug’s self-promotion and combinatorial therapy is demonstrated. The drug nanorods allowed for efficient intracellular delivery of the nucleic acid and the resultant autophagy inhibition in vitro and in vivo, which in turn sensitized the cancer cells to the anti-tumor nanorods. The developed carrier-free combinatorial therapy has overwhelmed benefits in the payload capacity and direct cytosol delivery of biological macromolecules. By using the approach, combining PTX with the autophagy inhibitor, miR-101, tumor growth can be suppressed with significantly higher efficiency than single-drug strategy. We believe the platform is generalized for combating against drug resistance and potently using the autophagy regulators in disease treatment.

## Electronic supplementary material

Below is the link to the electronic supplementary material.

Supplementary material 1 (PDF 2173 kb)
