# Supplementary material for: Drug Nanorod-Mediated Intracellular Delivery of microRNA-101 for Self-sensitization via Autophagy Inhibition
Source: Nanomicro Lett. 2019 Sep 25;11:82. doi: 10.1007/s40820-019-0310-0 (PMC7770860; doi:10.1007/s40820-019-0310-0)
Supplement: Supplementary file 1 — Supplementary material 1 (PDF 2173 kb) [file 40820_2019_310_MOESM1_ESM.pdf]

Supporting Information for

Drug Nanorod-Mediated Intracellular Delivery of microRNA-101 for Self-sensitization *via* Autophagy Inhibition

Xiaofei Xin[1], Xiaoqing Du[1], Qingqing Xiao[1], Helena S. Azevedo[2], Wei He[1, *], Lifang Yin[1, *]

[1]Department of Pharmaceutics, School of Pharmacy, China Pharmaceutical University, Nanjing, 210009, People's Republic of China

[2]School of Engineering and Materials Science, Institute of Bioengineering, Queen Mary, University of London, London, E1 4NS, UK

*Corresponding authors. E-mail: 1019940752@cpu.edu.cn (Lifang Yin); weihe@cpu.edu.cn (Wei He)

Supplementary Figures

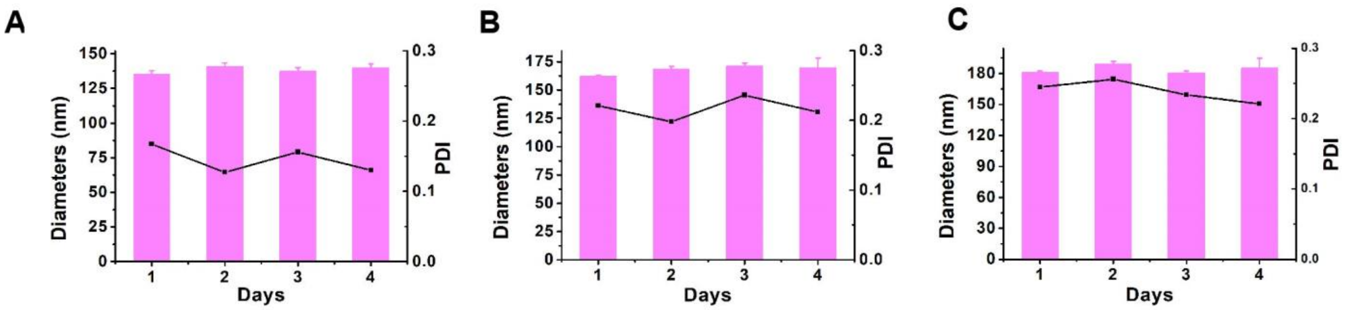

Fig. S1 The stability of (**A**) PNs, (**B**) PNplex and (**C**) HA-PNplex incubated in 10% serum at 37 °C for 4 days

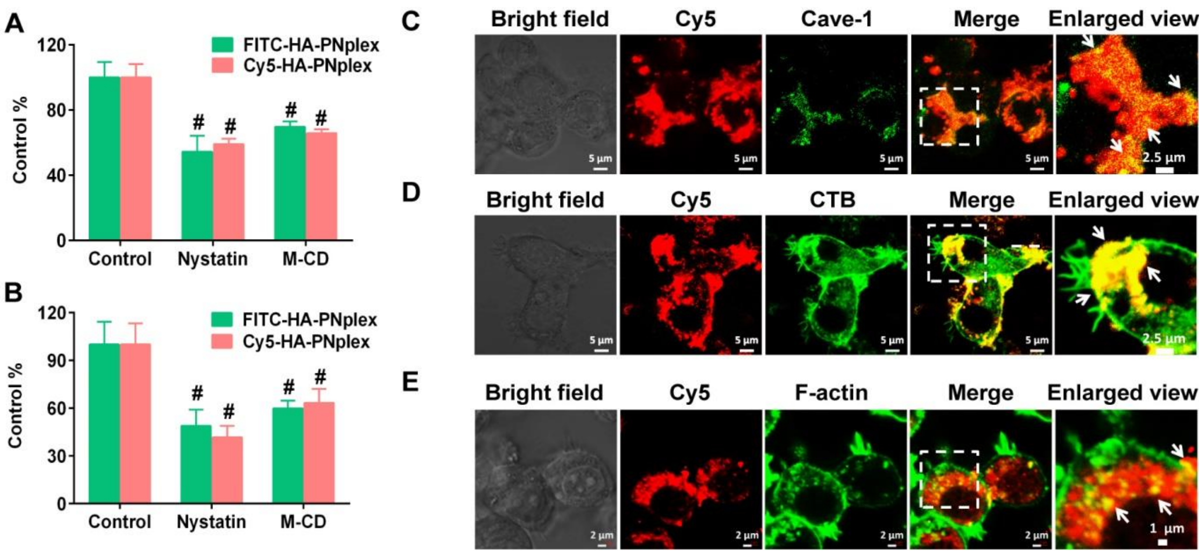

Fig. S2 Endocytic pathway and caveolae trafficking. Cellular uptake of FITC or Cy5-labeled HA-PNplex in (**A**) MCF-7 and (**B**) Caco-2 cells pretreated with nystatin or M-CD for 0.5 h at 37 °C at a FITC concentration of 2.5 μg mL^{-1} or Cy5-miR-101 at 100 nM. The fluorescence intensity was detected using FCM. $^{\#}P < 0.001$ versus the control *(n* = 5). (**C–E**) Caveolae-trafficking study. Co-localization of Cy5-HA-PNplex with Alexa Fluor 488-labeled (**C**) Cave-1 and (**D**) CTB and FITC-labeled (**E**) F-actin in Caco-2 cells was observed using CLSM post incubation at a Cy5 concentration of 100 nM for 3 h at 37 °C. Yellow fluorescence indicates the co-localization.

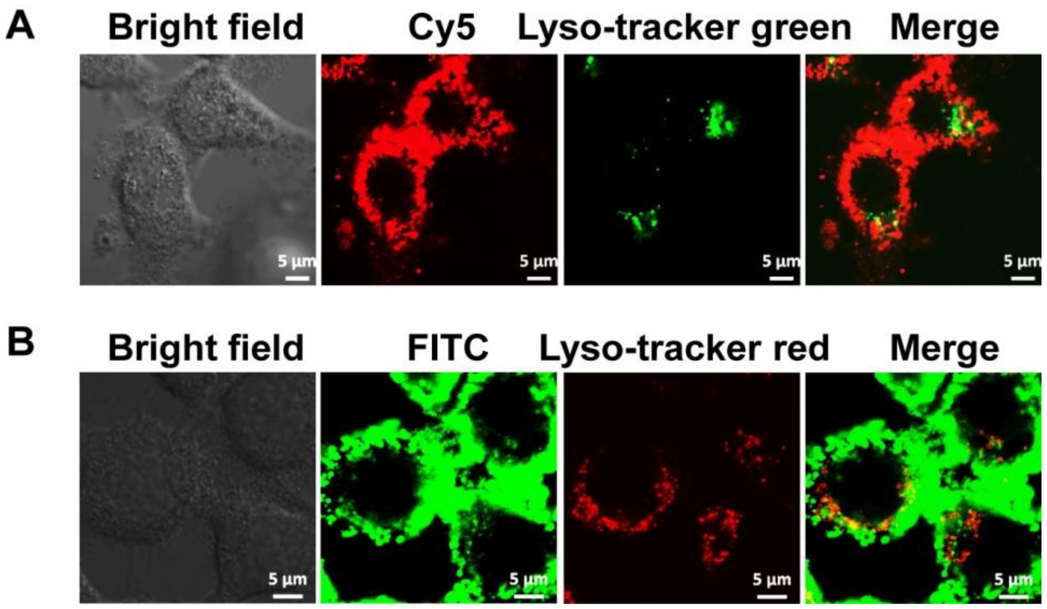

Fig. S3 Co-localization of (**A**) Cy5- or (**B**) FITC-labeled HA-PNplex with lysosomes in MCF-7 cells upon incubation with a FITC concentration of 2.5 μg mL^{-1} or Cy5-miR-101 concentration of 100 nM at 37 °C for 3 h

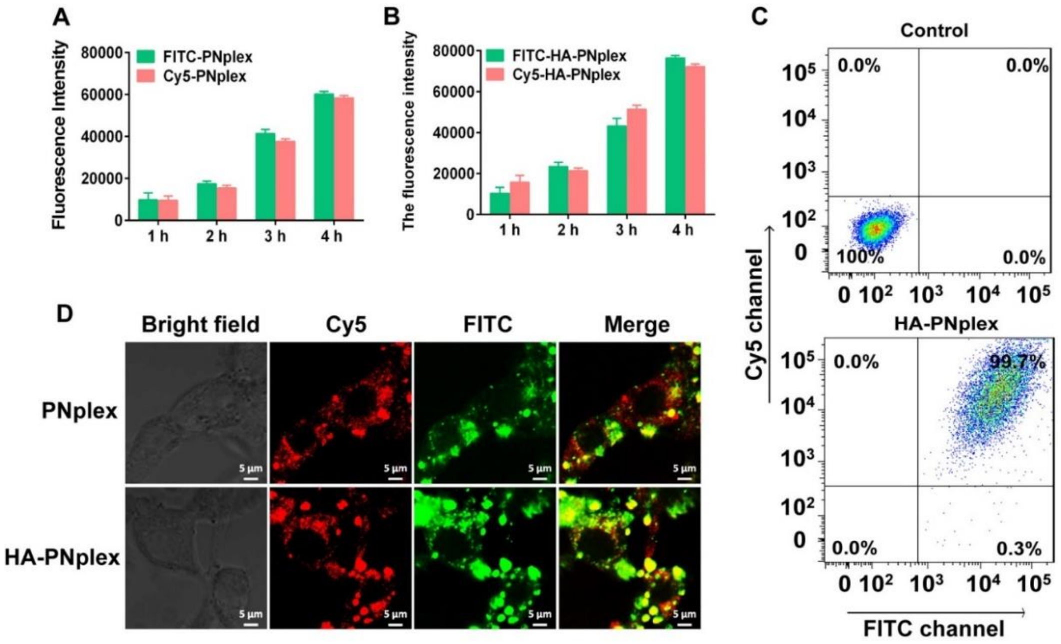

Fig. S4 Time-related cellular uptake of dual-labeled (**A**) PNplex and (**B**) HA-PNplex in MCF-7 cells after incubation at 37 °C with a FITC concentration of 2.5 μg mL^{-1} or Cy5-miR-101 concentration of 100 nM. The higher fluorescence intensity of HA-PNplex over PNplex indicated the targeting-ability of HA toward CD44-receptors. (**C**) Flow-cytometry images of the intracellular uptake of HA-PNplex. (**D**) Intracellular distribution of dual-labeled PNplex and HA-PNplex in MCF-7 cells after incubation at 37 °C for 3 h. Yellow spots indicate the integrity of the nanoparticles.

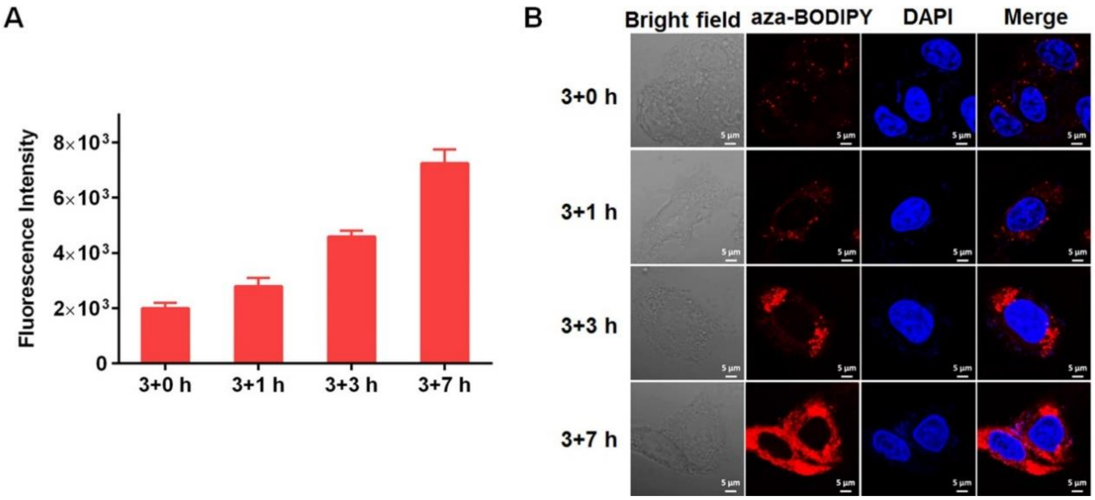

Fig. S5 Intracellular fate study. MCF-7 cells were cultured with aza-BODIPY labeled HA-PNplex at 37 °C for 3 h first using an aza-BODIPY concentration of 500 ng mL^{-1}, followed by serum-free media incubation for 0, 1, 3, 7 h at 37 °C. (**A**) Fluorescence intensity quantified by flow cytometry and (**B**) CLSM observation. When released in the cytoplasm the aza-BODIPY dye emits red fluorescence

owing to the aggregation in intracellular lipid-substrates. The red fluorescence indicates the release of the dye from the aza-BODIPY labeled HA-PNplex and, therefore, implies the disintegration of the nanoparticles.

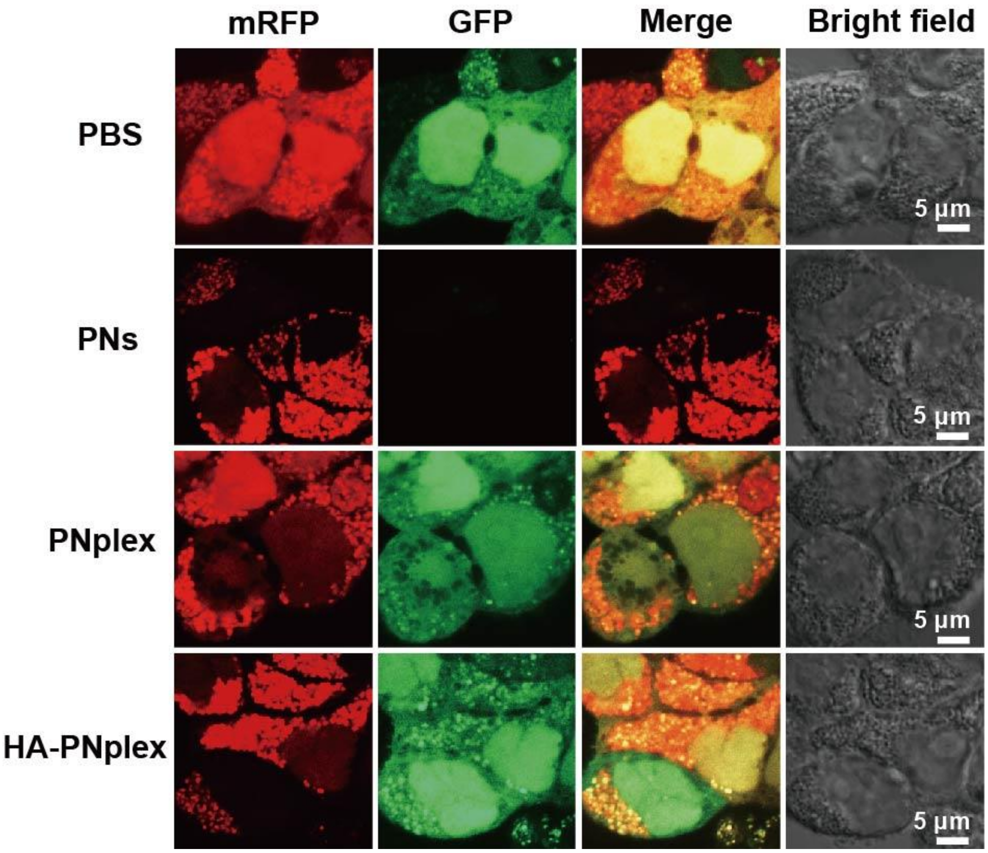

Fig. S6 Study of autophagy flux in mRFP/EGFP-MCF-7 cells incubated with 10 μg mL^{-1} PTX or 100 nM miR-101 for 24 h at 37 °C. The green fluorescence is negatively correlated with the fusion of autophagosomes and lysosomes. The yellow fluorescence indicates the absent fusion of autophagosomes and lysosomes.

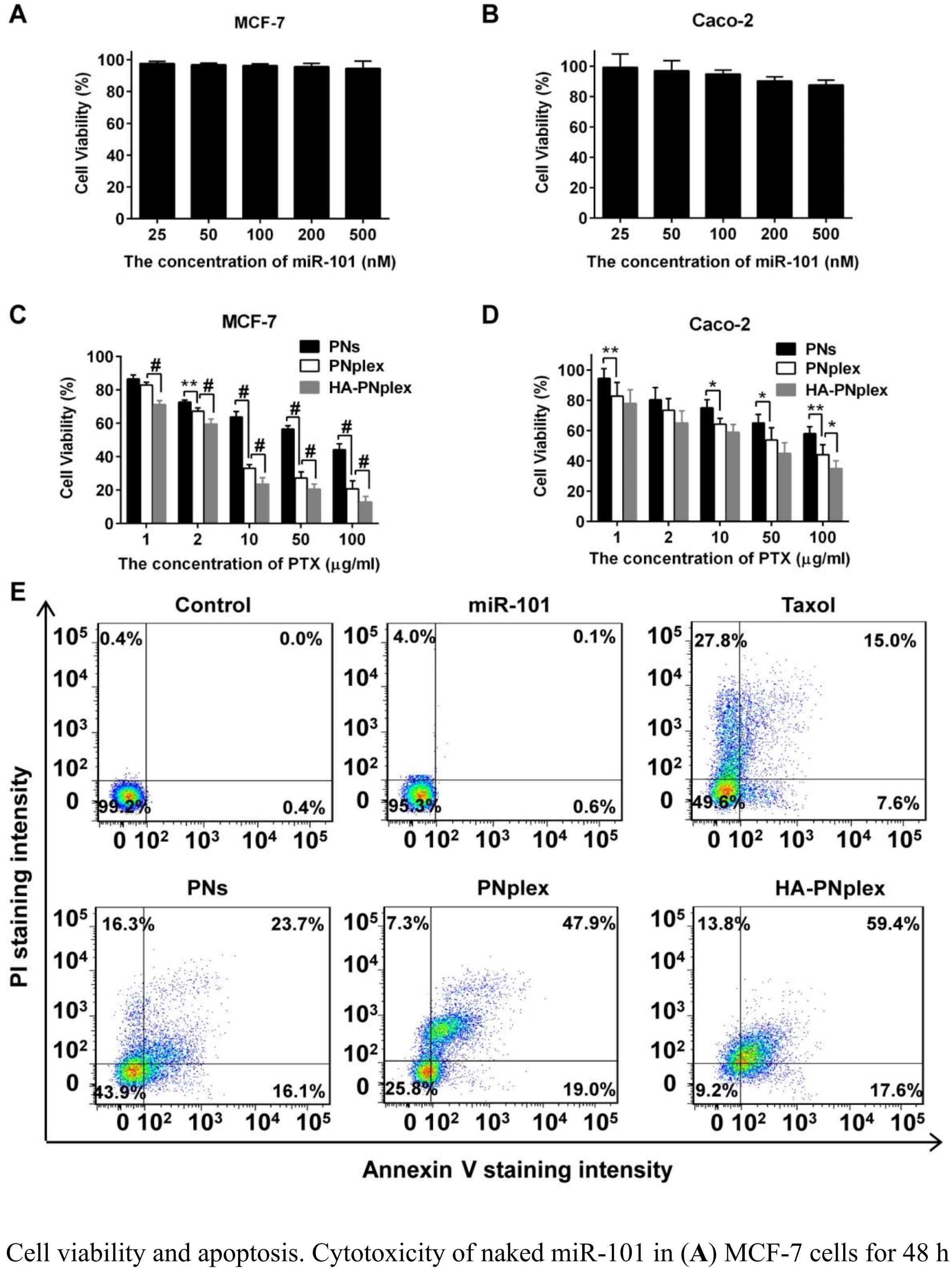

Fig. S7 Cell viability and apoptosis. Cytotoxicity of naked miR-101 in (**A**) MCF-7 cells for 48 h and (**B**) Caco-2 cells for 4 h at serial miR-101 concentrations at 37 °C. Cytotoxicity of the nanoparticles with various PTX concentrations after incubation at 37 °C for 48 h for (**C**) MCF-7 cells or 4 h for (**D**) Caco-2 cells ($n = 5$, $^{*}P < 0.05$, $^{**}P < 0.01$ and $^{\#}P < 0.001$). (**E**) Flow-cytometry images of cell apoptosis

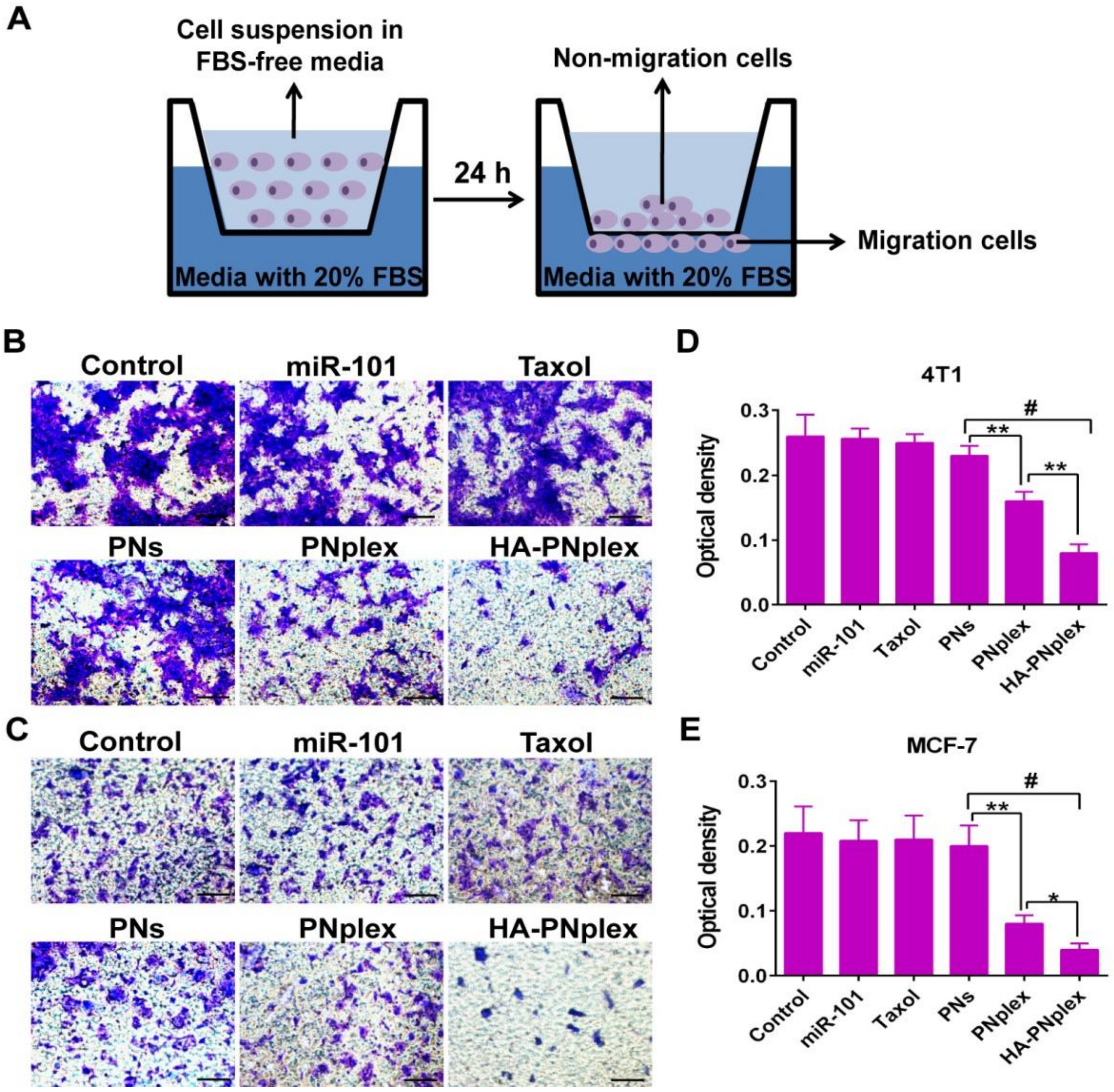

Fig. S8 *In vitro* anti-metastasis study. (**A)** Scheme of cell migration in the transwell chamber. Migration suppression of (**B**) 4T1 and (**C**) MCF-7 cell lines studied by a transwell chamber assay at 37 °C. Violet spots represent migrated cells. The control group corresponds to cells without treatment. The scale bar is 50 μm. Quantitative analysis of cell migration in (**D**) 4T1 and (**E**) MCF-7 cells (n = 3, $^{*}P < 0.05$, $^{**}P < 0.01$ and $^{\#}P < 0.001$ vs control)

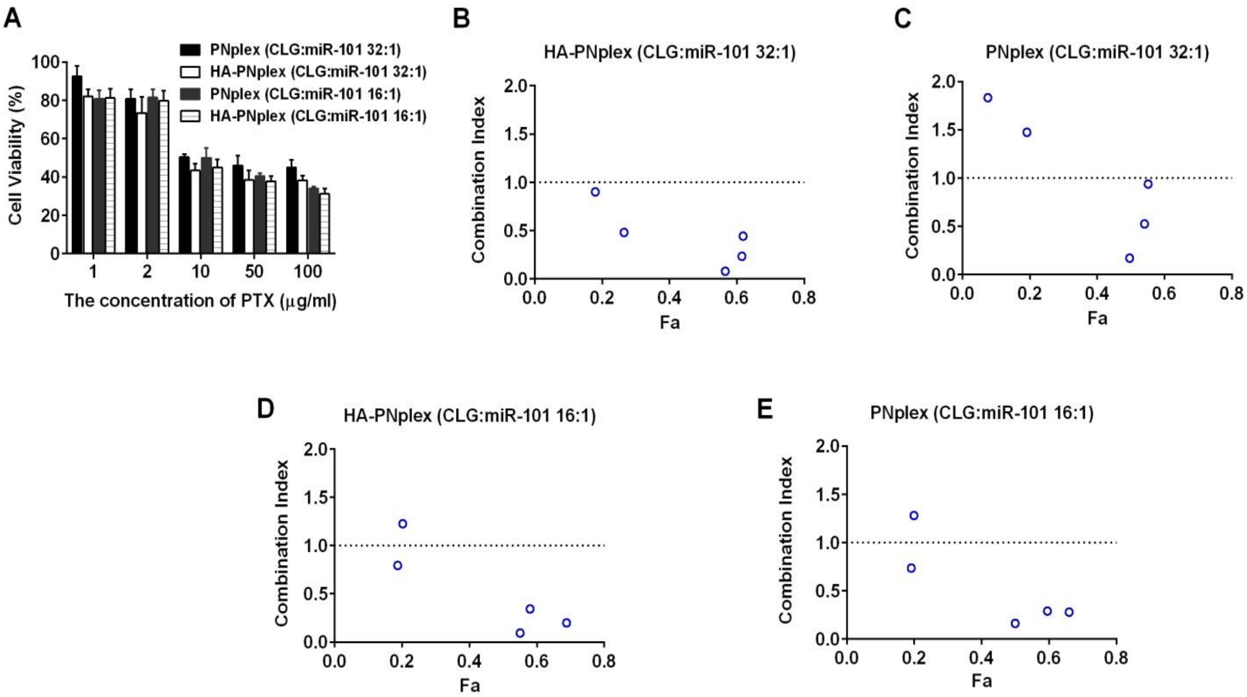

Fig. S9 Synergy of combinatorial therapy. (**A**) Cell viability upon exposure to PNplex and HA-PNplex at different CLG/miR-101 ratios with PTX concentration ranging from 1 to 100 μg mL^{-1}; Based on the cytotoxicity results, the coefficient of drug interaction (CDI) between the two drugs was calculated by the CompuSyn software. (**B–E**) Combination Index (*CI*) plot of above different formulations. *Fa* represents the fractional inhibition value. *CI* < 1, = 1, and > 1 indicates synergism, additive effect and antagonism, respectively.

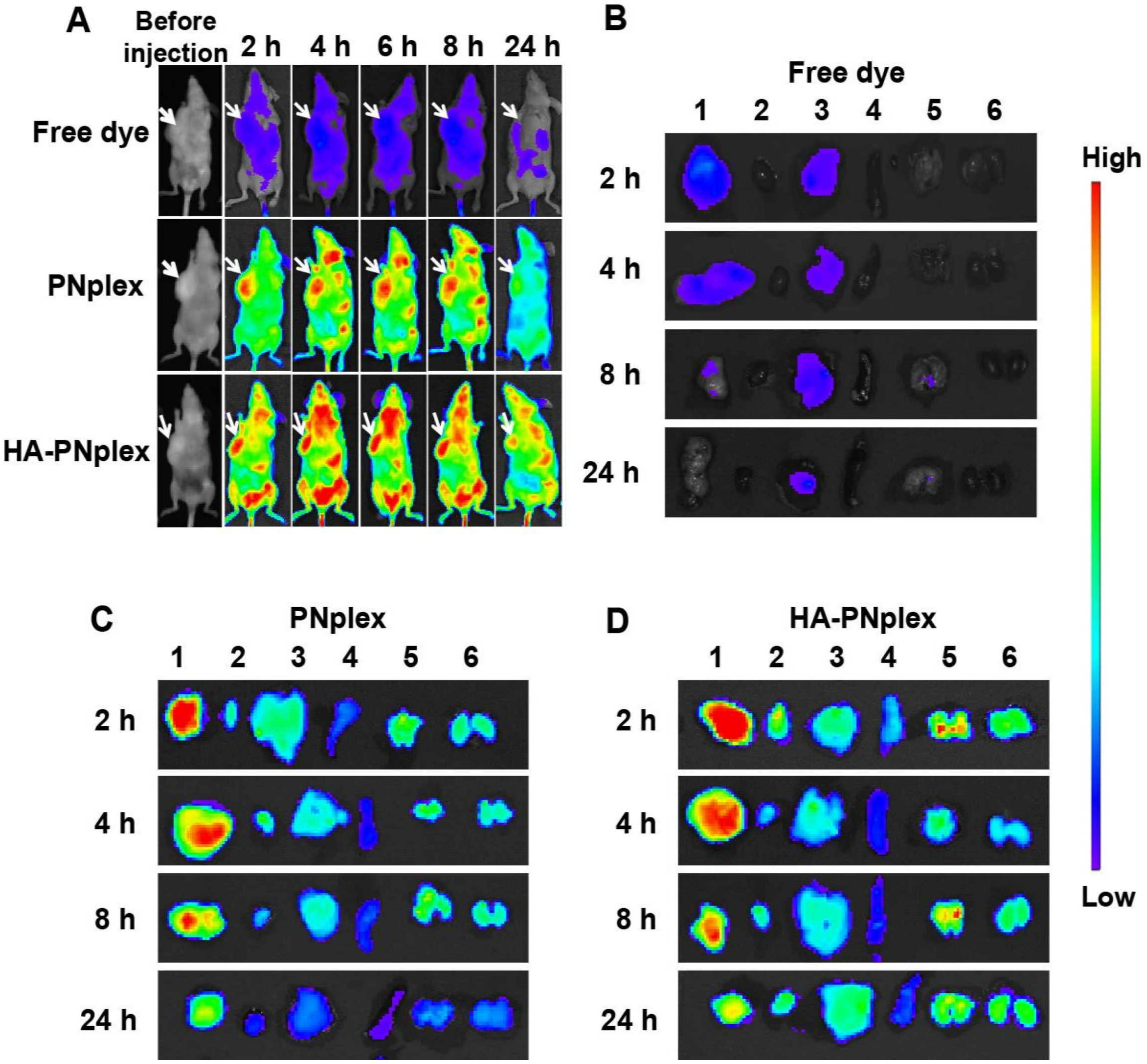

Fig. S10 Biodistribution and tumor targeting studies. (**A**) *In vivo* imaging of MCF-7 tumor-bearing nude Balb/C mice (pointed by the white arrow) at predetermined time points after injection of IR 783-labeled nanoparticles (red fluorescence) at an IR783 dose of 2.5 mg/kg based on the body's weight. Free IR783 was used as control. (**B–D**) *Ex vivo* fluorescence images of tissues including tumor (1), heart (2), liver (3), spleen (4), lung (5) and kidneys (6) collected at 2 h, 4 h, 8 h, and 24 h following injection

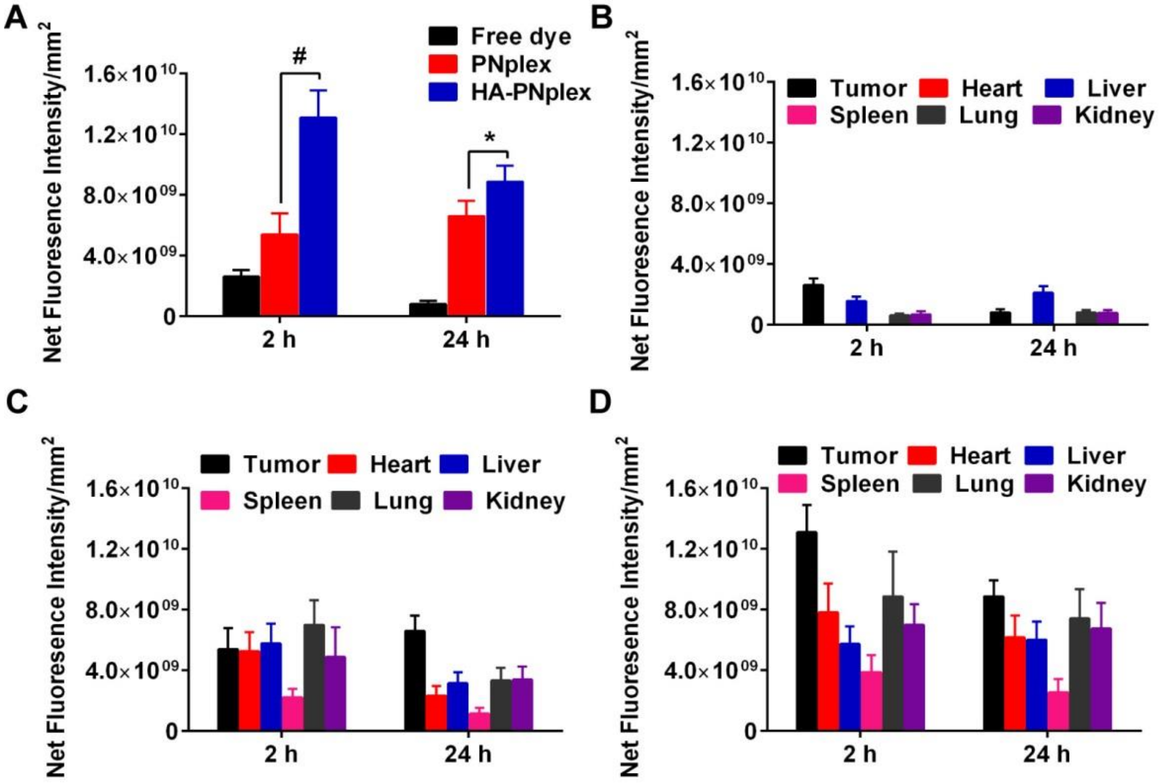

Fig. S11 (**A**) Quantification assay of accumulation of IR783-labeled nanoparticles in tumor at predetermined time points following injection at a IR783 dose of 2.5 mg kg^{-1}, according to the animal's weight ($n = 3$, $^{*}P < 0.05$ and $^{\#}P < 0.001$). Quantified accumulation of (**B**) free IR783, (**C**) IR783-PNplex and (**D**) IR783-HA-PNplex in tissues at 2 and 24 h post injection

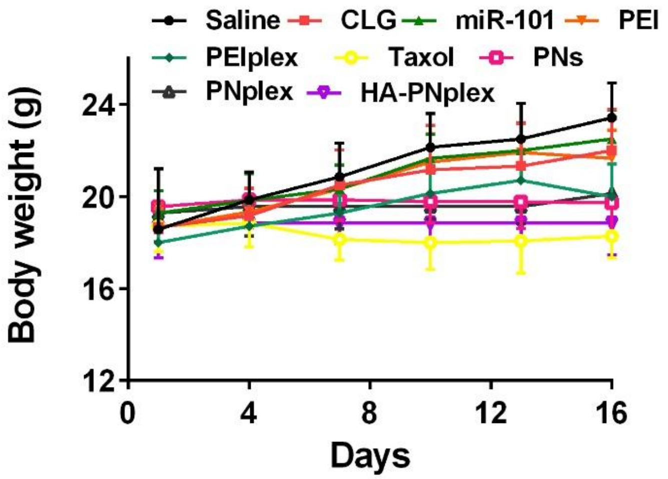

Fig. S12 Body weights in antitumor efficacy ($n = 6$)

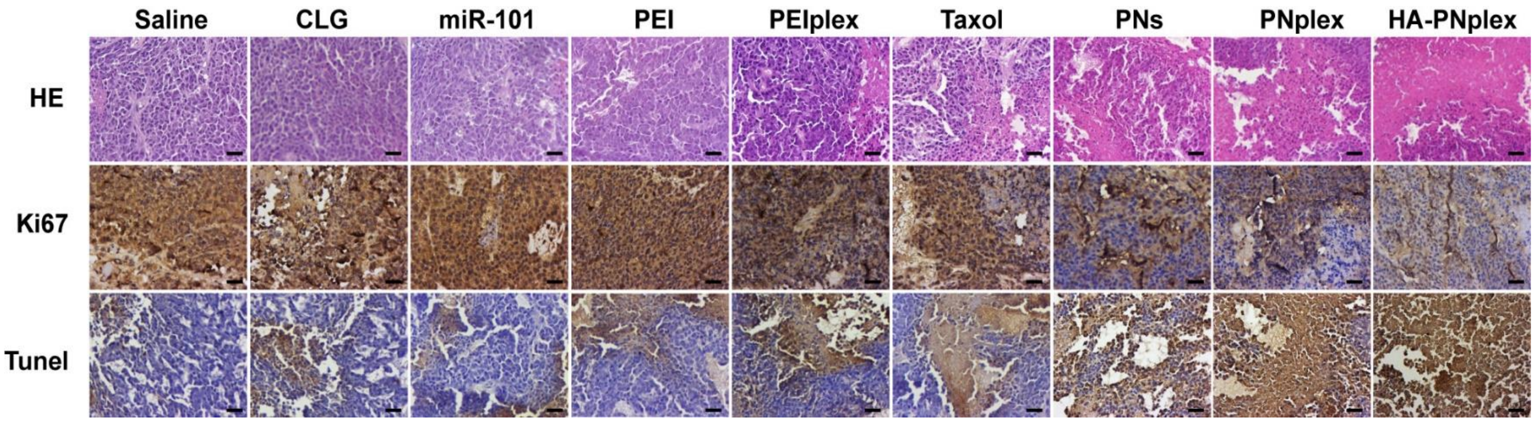

Fig. S13 H&E staining, Ki67 and TUNEL analysis of the tumor specimens. The brown-stained cells represent positive cells in the Ki67 and TUNEL assay. Nuclei are stained blue, while extracellular matrix and cytoplasm are stained red in H&E analysis. The absence of nuclei represents the necrosis of tumor cells. The scale bar is 20 μm.

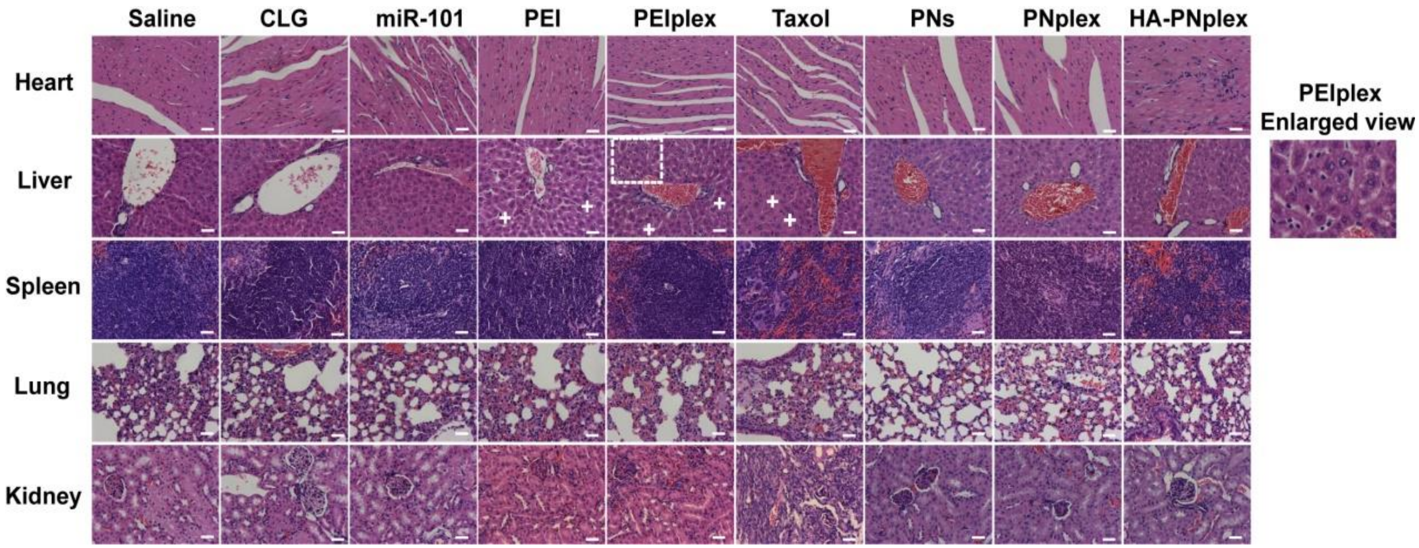

Fig. S14 Representative histological images from retrieved tissues. Formulations were administered to MCF-7 tumor-bearing nude Balb/C mice *via* tail vein injections every 3 days at a PTX dose of 10 mg kg^{-1} or a miR-101 dose of 1 mg kg^{-1}, according to the animal's body

weight. The tissues were collected at day 17 post repeat dosing. Nuclei are stained blue while extracellular matrix and cytoplasm are stained red in H&E analysis. “+” represents the positive area. The scale bar is 20 µm. The enlarged view of PEIplex-treated group is the positive area displayed as spotty or lytic necrosis and hydropic degeneration induced by inflammation.

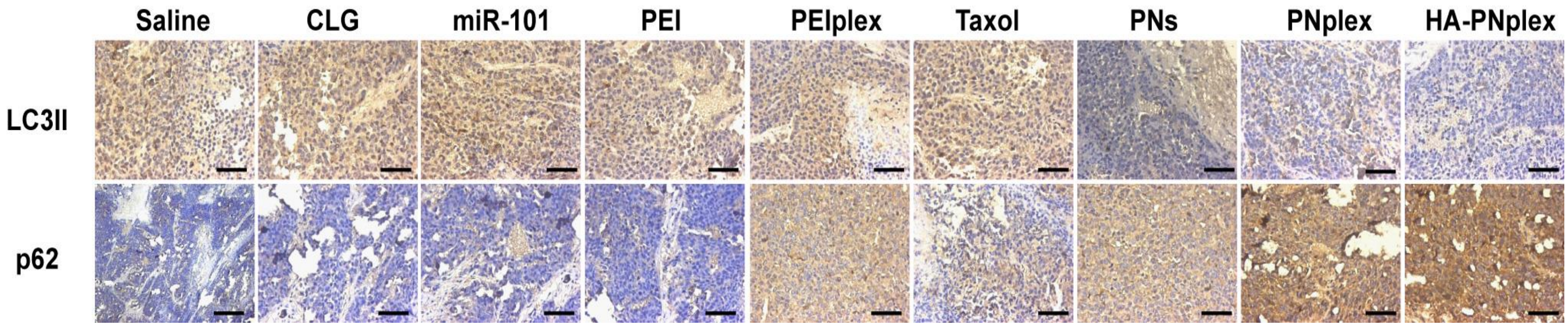

Fig. S15 *In vivo* inhibition of autophagy flux. Immunohistochemistry images of LC3II and p62 in tumor specimens. The brown-stained cells represent the positive cells in the LC3II and p62 IHC assay and nuclei are stained blue. The scale bar is 20 µm.
